# TFEB Probably Involved in Midazolam-Disturbed Lysosomal Homeostasis and Its Induced β-Amyloid Accumulation

**DOI:** 10.3389/fnhum.2019.00108

**Published:** 2019-05-21

**Authors:** Dan Cheng, Qilian Tan, Qianyun Zhu, Jiqian Zhang, Xiaoyu Han, Panpan Fang, Weilin Jin, Xuesheng Liu

**Affiliations:** ^1^Department of Anesthesiology, First Affiliated Hospital of Anhui Medical University, Hefei, China; ^2^Key Laboratory for Thin Film and Microfabrication Technology of Ministry of Education, Institute of Nano Biomedicine and Engineering, Department of Instrument Science and Engineering, School of Electronic Information and Electronic Engineering, Shanghai Jiao Tong University, Shanghai, China

**Keywords:** emotion, β-amyloid, lysosome, TFEB, midazolam

## Abstract

Alzheimer’s disease (AD) is one of the most common neurodegenerative diseases, and β-amyloid (Aβ) plays a leading role in the pathogenesis of AD. The transcription factor EB (TFEB), a main regulating factor of autophagy and lysosome biosynthesis, is involved in the pathogenesis of AD by regulating autophagy-lysosomal pathways. To date, the choice of anesthetics during surgery in patients with neurodegenerative diseases and evaluation of the effects and underlying mechanisms in these patients have rarely been reported. In this study, the HEK293-APP cells overexpressing APP and Hela cells were used. The cells were treated with midazolam at different concentrations and at different times, then lysosomes were stained by lysotracker and their morphology was observed under a fluorescence microscope. The number and size of lysosomes were analyzed using the ImageJ software. The levels of TFEB in the nucleus and APP-cleaved intracellular proteins were detected by nuclear separation and Western Blot. Finally, ELISA was used to detect the levels of Aβ40 and Aβ42 in the cells after drug treatment. We found that 30 μM midazolam decreased the number of lysosomes and increased its size in HEK293 and HeLa cells. However, 15 μM midazolam transiently disturbed lysosomal homeostasis at 24 h and recovered it at 36 h. Notably, there was no significant difference in the extent to which lysosomal homeostasis was disturbed between treatments of different concentrations of midazolam at 24 h. In addition, 30 μM midazolam prevents the transport of TFEB to the nucleus in either normal or starved cells. Finally, the intracellular C-terminal fragment β (CTFβ), CTFα, Aβ40 and Aβ42 levels were all significantly elevated in 30 μM midazolam-treated HKE293-APP cells. Collectively, the inhibition of TFEB transport to the nucleus may be involved in midazolam-disturbed lysosomal homeostasis and its induced Aβ accumulation *in vitro*. The results indicated the risk of accelerating the pathogenesis of AD by midazolam and suggested that TFEB might be a candidate target for reduction of midazolam-dependent neurotoxicity.

## Introduction

Alzheimer’s disease (AD) is one of the most common neurodegenerative diseases, mainly characterized by progressive learning and memory dysfunction, and many of the patients with AD exhibit abnormal emotions (Mirakhur et al., 2004; Potter and Steffens, 2007). Extracellular β-amyloid (Aβ) deposition, intracellular neurofibrillary tangles and a decreased number of synapses and neurons are the dominant pathological features (Laferla et al., 2007; Ubhi and Masliah, 2013) and among them, Aβ received particular attention. The amyloid precursor protein APP is first hydrolyzed by β-secretase and α-secretase to produce the corresponding C-terminal fragment β (CTFβ) C99 and shorter C83 (CTFα), and then CTFβ is further cleaved by γ-secretase to produce Aβ and other fragments (Vassar et al., 2009; De Strooper and Annaert, 2010). Aβ40 and Aβ42 are the main components of long-chain Aβ *in vivo* (Tahmasebinia and Emadi, 2017). Multiple studies have shown that Aβ is closely related to apathy-like behavior, anxiety-like behavior and depression (Wu et al., 2015; Zare et al., 2015; Souza et al., 2016). There are many clinical studies showing that anesthetics can induce postoperative delirium and postoperative cognitive dysfunction in surgical patients (Bilotta et al., 2010; Hussain et al., 2014). Numerous laboratory studies have revealed that inhaled anesthetic sevoflurane and isoflurane could promote the processing of APP, Aβ production and accelerate the progression of AD-related pathological development (Dong et al., 2009; Xie and Xu, 2013; Zhang et al., 2017). However, the effects and mechanisms of intravenous anesthetics on AD are rare.

As an important organelle for intracellular constituent degradation, signal transduction, cellular secretion, plasma membrane repair and energy metabolism, lysosomes are closely associated with neurodegenerative diseases *via* clearance of damaged organelles or aggregated proteins that can cause disease (Settembre et al., 2013b). Lysosomal dysfunction can lead to abnormal protein degradation disorders and deposition, which may cause neurodegenerative diseases (Nixon et al., 2000; Zhang et al., 2009). Abnormal lysosome accumulation is one early histological change in AD patients (Cataldo et al., 1994, 2000; Nixon et al., 2000), and enhancement of lysosomal function can reduce the amyloid deposition and improve the cognitive function in the mouse model of AD (Kawarabayashi et al., 1997; Shie et al., 2003; Langui et al., 2004). Besides, our previous study revealed that inhaled anesthetic sevoflurane could impair autophagic degradation, which depends on lysosomal function, and accelerates the pathological progress of AD in APP/PS1 mouse (Geng et al., 2018). Our published research has showed that midazolam could increase mutant huntingtin protein levels (Zhang et al., 2018). However, the underlying mechanisms of how anesthetics impact on lysosome function is unknown.

The transcription factor EB (TFEB) is a major regulator of lysosomal biosynthesis, which is coordinated by driving autophagy and expression of lysosomal genes (Settembre et al., 2011). TFEB exists in the cytoplasm in the form of inactive phosphorylation under the physiological condition (Kim et al., 2016). In the case of lysosomal abnormalities or starvation, TFEB translocates from the cytoplasm to the nucleus and performs its function as a transcription factor (Settembre et al., 2013a). Xiao and Zhang’s study has demonstrated that TFEB can regulate production of autophagosomes and degradation of lysosomes by the autophagy-lysosomal pathway in the mouse model of AD, which accelerates Aβ and amyloid plaques clearance (Xiao et al., 2014, 2015; Zhang and Zhao, 2015) and improves the cognitive function of mouse (Zhang and Zhao, 2015). Increasing evidence has revealed that some drugs attenuate amyloid plaque pathology by regulating TFEB (Bao et al., 2016; Chandra et al., 2018), but there are few studies on the regulation of TFEB by anesthetics.

Midazolam is a commonly used intravenous anesthetic for sedation and balanced general anesthesia during surgery. Our previous study has shown that midazolam could impair the autophagic degradation by downregulating the lysosomal aspartyl protease cathepsin D levels. In this work, we found that 30 μM midazolam decreased the number of lysosomes and increased its size in HEK293 and HeLa cells. Midazolam could also prevent TFEB transport to the nucleus, which may account for the impaired lysosomal homeostasis. Finally, the intracellular Aβ levels were elevated in midazolam treated HKE293-APP cells. These results revealed the risk of accelerating the pathogenesis of AD by midazolam and implied the probable mechanism of anaesthetic-induced abnormal emotion in surgery patients.

## Materials and Methods

### Antibodies and Agents

Lyso-Tracker Red (1:10,000; DND-99) was purchased from invitrogen; anti-TFEB antibody (1:800; 13,372-1-AP) was purchased from proteintech; anti-H3 antibody (1:1,500; 17,168-1-AP) was purchased from Abcam, USA; mouse anti-Aβ antibody (1:1,000 dilution) was purchased from Abcam, USA; mouse anti-β-actin antibody (1:1,000 dilution) was purchased from Abcam, USA; mouse monoclonal antibodies used were agonist Aβ (1:10,000) and β-actin (1:10,000); rabbit monoclonal antibodies used were against TFEB (1:5,000) and H3 (1:10,000); BCA protein quantification kit was purchased from China Biyuntian Biotechnology Co., Ltd.; Aβ40 and Aβ42 ELISA kits were purchased from CUSABIO, China.

### Cell Culture

Cells were cultured at 37°C with 5% CO_2_ in Dulbecco’s modified Eagle’s medium supplemented with 10% fetal bovine serum (FBS). HEK293 cells are primary embryonic human kidney cells, HEK293-APP are APP-overexpressed HEK293B cells, and these cells were kindly provided by WJ in Shanghai Jiao Tong University. HeLa cells are human cervical cancer cell lines, and they were kindly provided by Professor Longping Wen in the University of Science and Technology of China. Nutrient starvation assays were performed in the presence of Earle’s Balanced Salt Solution (EBSS).

### Lysosomal Staining

For LysoTracker Red DND-99 assay, Cells were seeded in a 24-well culture plate at a density of 5 × 10^4^ cells per well, incubated in 37°C, 5% CO_2_ for 24 h and then treated with midazolam for different times and washed twice in PBS. Next, the cells were incubated in medium containing 1 μM LysoTracker Red DND-99 (Invitrogen, L-7528) dye for 10 min. Cells were washed twice in PBS and observed under an LSM 710 confocal microscope (Carl Zeiss AG, Oberkochen Germany). The size and number of lysosomes were measured by ImageJ software using its “analyze particle” analysis tool with default image/adjust/threshold settings. For lysosomal morphology and size, as well as the effect size between the two groups were calculated using Ellis (2010), “Effect Size FAQs,” website.

### Co-location Detection

To explore the relationship between midazolam and TFEB translocation, the live cell imaging analysis was carried out. After being treated with or without 30 μM midazolam for 24 h and starved for 4 h, EGFP-TFEB/HeLa Cells were washed twice in PBS, then incubated with DAPI (blue) for 10 min, the cellular fluorescence was observed by confocal microscopy (Carl Zeiss AG, Oberkochen Germany). The HeLa EGFP-TFEB cell lines were kindly provided by professor Wen Longping, which expressed strong green fluorescence under a fluorescence microscope.

### Nuclear and Cytoplasm Separation

The sucrose buffer [1 M sucrose, 0.1 M CaCl_2_, 1 m magnesium acetate, 250 × 10^−3^ M ethylenediaminetetraacetic acid (EDTA), 100 × 10^−3^ M dithiothreitol (DTT), and 100 × 10^−3^ M phenylmethylsulfonyl fluoride (PMSF)] was used to separate nuclear and cytoplasm fraction. Briefly, cells were collected from a 24-well cell culture plate after treatment and washed by PBS, and then 100–200 μL sucrose NP-40 (0.5% NP-40 in sucrose buffer) buffer was added. Those samples were put on ice for 15 min and centrifuged for 10 min at 1,000× *g*. The supernatant was the cytoplasm fraction. In contrast, the precipitation was the nuclear fraction and washed by sucrose buffer before adding cell lysis buffer and boiling.

### Western Blot Analysis

Cells were lysed with sample buffer and boiled for 10 min. Proteins were separated by sodium dodecyl sulfate polyacrylamide gel electrophoresis and were transferred onto nitrocellulose membranes. The membranes were incubated with the primary antibody at 4°C overnight and the second antibody for 1 h at 37°C. Membranes were incubated with the ECL kit and visualized using a chemiluminescence instrument (ImageQuant LAS 4000, GE Healthcare, Little Chalfont, UK).

### Enzyme-Linked Immunosorbent Assay

The cells were homogenized in PBS followed by RIPA buffer [50 mM Tris-HCl, 150 mM NaCl, 1% Triton X-100, 0.1% SDS, and 1× protease inhibitor (Xiao et al., 2014)]. The concentrations of Aβ40 and Aβ42 intracellular were detected by a human-specific ELISA kit (CUSABIO, China), according to the manufacturer’s instructions.

### Experimental Grouping

(1) For lysosomal homeostasis detection, HEK293 and Hela cells were divided into two groups: 15 μM and 30 μM midazolam treatment groups. At 0, 24 h and 36 h, the size and morphology of the lysosomes were observed; (2) for TFEB levels detection, HEK293 and Hela cells were divided into two groups: 15 μM and 30 μM midazolam treatment groups. Detect the levels of TFEB at different times (0 h, 24 h, 36 h) in the nucleus; (3) for TFEB levels detection in the case of starvation with or without 30 μM midazolam, HEK293 and Hela cells were divided into four groups: control, midazolam, starvation and midazolam+starvation. Detect the level of TFEB in the nucleus; and (4) for Aβ levels detection, HEK293 cells were divided into control and 30 μM midazolam treatment group. Detect the C83, C99 levels.

### Statistical Analysis

All data are shown as Mean ± SEM. Data were analyzed by two-tailed Student’s *t*-test for detecting significant differences between two groups. For lysosomal homeostasis detection, the lysosomal size and number were analyzed by one-way ANOVA followed by *post hoc* Tukey’s test. The Western Blot results were analyzed by one-way ANOVA, followed by Tukey’s *Post hoc* test or two-tailed Student’s *t-test*. For Aβ40 and Aβ42 detection using ELISA, the results were analyzed by two-tailed Student’s *t-test*. Differences were considered statistically significant at *p < 0.05 and **p < 0.01, ***p < 0.001.

## Results

### Midazolam Impair Lysosomal Homeostasis

**Figure 1:**
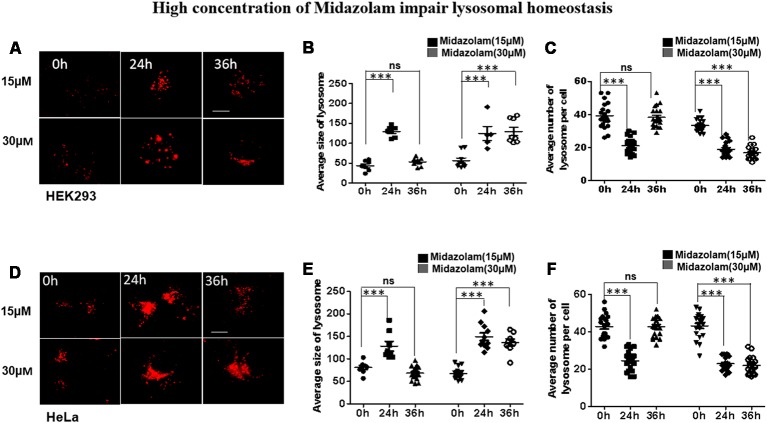
Midazolam disturbs lysosomal homeostasis. HEK293 and Hela cells were treated with 15 μM or 30 μM midazolam for 0–36 h. The size and morphology of the lysosomes were observed under a microscope. **(A,D)** Lysosomal staining pattern of HEK293 cells and HeLa cells treated with 15 μM or 30 μM midazolam at 0 h, 24 h, and 36 h, respectively. **(B,E)** Statistical results of the average size of lysosomes in HEK293 cells and HeLa cells, respectively. **(C,F)** Statistical results of the average number of lysosomes per HEK293 and HeLa cell, respectively. For lysosomal number and lysosomal size statistics, at least 30 cells per group were counted. Scale bar = 2 μM (Mean ± SEM. ***p < 0.001, ns, no significant).

To evaluate the effect of midazolam on lysosomes, HEK293 and HeLa cells were treated with different concentrations of midazolam for 24 h or 36 h. After 15 μM midazolam treatment, the number (*p* < 0.001, effect sizes: 2.820) and size (*p* < 0.001, effect sizes: 6.586) of lysosomes were transiently decreased and became larger at 24 h compared with 0 h, respectively. The above changes were counteracted at 36 h (*p* > 0.5, effect sizes: 0.036; 0.824; Figures 1A–C). However, 30 μM midazolam continuously decreased lysosome number (*p* < 0.001, effect sizes: 4.480) and swelled lysosomes (*p* < 0.001, effect sizes: 2.890) until 36 h (Figures 1A–C). What’s more, the same experiment was carried out in Hela cells, and the results were similar to those of HEK293 cells (Figures 1D–F), at 15 μM midazolam, the number (*p* < 0.001, effect sizes: 3.115) and size (*p* < 0.001, effect sizes: 2.035) of lysosomes temporarily decreased and became larger at 24 h, and these changes were offset at 36 h (*p* > 0.5, effect sizes: 0.017; 0.896), but 30 μM midazolam continued to reduce the number of lysosomes (*p* < 0.001, effect sizes: 3.70) and lysosomes expand (*p* < 0.001, effect sizes: 3.71) until 36 h. Notably, the size and number of lysosomes had no significant difference under different concentrations of midazolam treatment at 24 h, indicating the similar extent of damage to the lysosomal homeostasis by midazolam (Supplementary Figure S1).

### Lysosome Homeostasis Disruption Accompanied by Failed Transportation of TFEB

**Figure 2:**
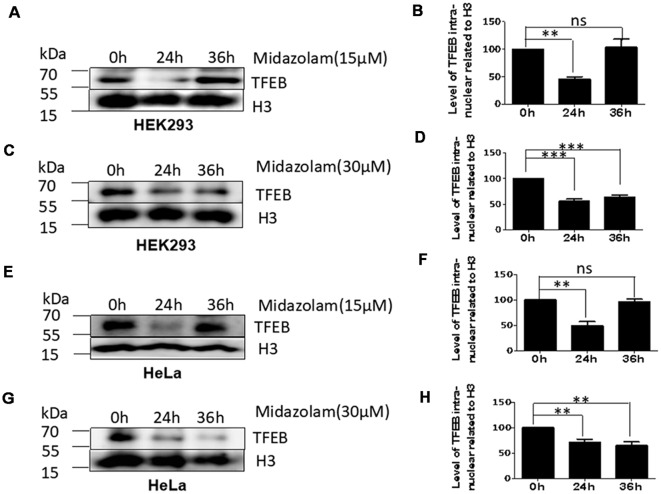
Lysosome homeostasis disruption accompanied by TFEB fails to transport to the nucleus. After treatment with 15 μM or 30 μM midazolam, the levels of TFEB at different times in the nucleus were examined. **(A,C)** Western Blot results of nuclear TFEB level in HEK293 cells. **(B,D)** Statistical results of **(A,C)**, respectively. **(E,G)** Western Blot results of nuclear TFEB level in HeLa cells. **(F,H)** Statistical results of **(B,D)**, respectively (Mean ± SEM. *N* = 3. **p < 0.01, ***p < 0.001, ns: no significant).

As TFEB is the major regulator of lysosome biosynthesis, the levels of TFEB in the nucleus was detected in midazolam-treated cells. The results showed that nuclear TFEB levels were reduced at 24 h in both HKK293 and HeLa cells treated with 15 μM midazolam compared with 0 h, but it was recovered at 36 h (Figures 2A,B,E,F). However, the nuclear TFEB continued to decrease until 36 h after 30 μM midazolam treatment (Figures 2C,D,G,H). These data were consistent with changes in the size and number of lysosomes previously found (Figures 1B,C,E,F), indicating a relationship between lysosomal homeostasis disruption and TFEB transportation failure.

### Midazolam Prevents TFEB From Transport to the Nucleus

**Figure 3:**
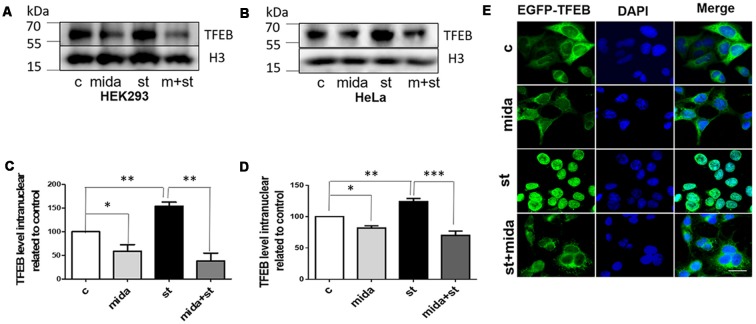
Midazolam prevents TFEB transport to the nucleus in the starved cell. Starvation 4 h as a positive control, is known to facilitate the intervention of TFEB into the nucleus. Before starved for 4 h, cells were pretreated with or without 30 μM midazolam for 24 h. **(A,B)** Western Blot results of nuclear TFEB levels in HEK293 cells and HeLa cells, respectively. **(C,D)** Statistical results of nuclear TFEB levels related to H3 levels in HEK293 cells and HeLa cells, respectively. **(E)** Representative fluorescent pictures of GFP-TFEB/HeLa cells nucleus translocation. The nucleus was stained by DAPI. Scale bar = 20 μM. C Control, Mida: Midazolam, St: Starvation (Mean ± SEM. *N* = 3. *p < 0.05, **p < 0.01, ***p < 0.001, ns, no significant).

In starvation condition, TFEB will transport from cytoplasm to nucleus to upregulate lysosomal function and autophagy, and then provide enough nutrients for the cells to survive (Settembre et al., 2011). To further verify whether midazolam could prevent TFEB transportation, nuclear TFEB levels were tested in starved cells with or without 30 μM midazolam treatment. Indeed, the TFEB levels were elevated in starved HEK293 cells. To the contrary, it was reduced in starved cells that were pretreated with midazolam (Figures 3A,C). Besides, the same results were observed in HeLa cells (Figures 3B,D), the levels of TFEB increased in HeLa cells for the treatment of starvation. Conversely, it was decreased in starved cells pretreated with midazolam. Furthermore, HeLa cells overexpressing GFP-TFEB were used to confirm the inhibition of TFEB translocation by midazolam. In starved HeLa cells, most of the GFP-tagged TFEB entered the nucleus, however, it was few in midazolam pre-treated starved cells (Figure 3E). These results demonstrated that 30 μM midazolam could indeed block TFEB from entering the nucleus. In this case, once lysosomes were disrupted by midazolam, it would not be repaired.

### Midazolam Induces Aβ Accumulation

**Figure 4:**
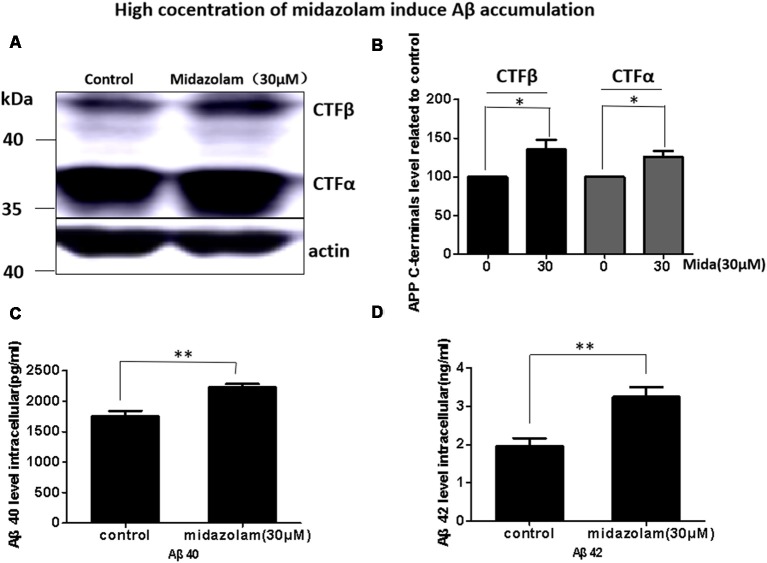
Midazolam triggers the accumulation of Aβ. HEK293-APP cells were treated with 30 μM midazolam for 36 h. **(A)** Western Blot results of C83, C99 levels. **(B)** Statistical results of **(A)**. **(C,D)** ELISA results of intracellular Aβ40 and Aβ42 levels (Mean ± SEM. *N* = 3. *p < 0.05, **p < 0.01).

Aβ has been shown to be neurotoxic and plays a leading role in the pathogenesis of AD. The lysosome is an important organ required for Aβ degradation (Xiao et al., 2014; Aguzzi and Haass, 2003; De Kimpe et al., 2013). However, 30 μM midazolam not only impaired lysosomal homeostasis but also had an impact on lysosome biogenesis regulator TFEB, thus, we assume that midazolam might change the metabolism of Aβ. Therefore, the level of APP cleavage products C83, C99, Aβ40 and Aβ42 were further detected in APP-overexpressed HEK293 cells. Indeed, the results showed that the contents of C83, C99 (Figures 4A,B), intracellular Aβ40 and Aβ42 (Figures 4C,D) were all elevated after midazolam treatment.

## Discussion

In this study, we found that the clinically used anesthetic sedative midazolam increased intracellular Aβ levels, suggesting that midazolam may promote the pathological process of AD. Additionally, our previous studies have also shown that midazolam increases intracellular mutant huntingtin protein, revealing its risk of accelerating the pathogenesis of polyglutamine diseases (Zhang et al., 2018). These results suggested that midazolam might have the ability to trigger the aggregation of neurotoxic proteins, and therefore, midazolam may not be the anesthetic of choice for use in patients with neurodegenerative disease.

Notably, midazolam-induced accumulation of neurotoxic protein aggregates was always accompanied by impaired lysosomes/autolysosomes function. In this article, we demonstrate that midazolam destroys lysosomal homeostasis primarily in HEK293 cells which is commonly used instrumental cells with high transfection efficiency and convenient operation in the study of molecular mechanism. They are also often used to study the pathogenesis of Alzheimer’s disease. HT22 is hippocampal neuronal cell lines of mouse. And we further test the inhibitory effect of midazolam on lysosomal homeostasis in HT22 cells, the result is the same as HEK293 cells (Supplementary Figure S2). Some other intravenous anesthetics have also been reported to impair lysosomes. Ren found that prolonged use of the anesthetic propofol increased the pH of the lysosome and destroyed the lysosomal function (Ren et al., 2017). Propofol could also induce lysosomal membrane permeabilization (LMP), loss of mitochondrial transmembrane potential (MTP) and caspase-dependent apoptosis (Hsing et al., 2012). Although our previous work had also shown that midazolam could disrupt the autophagic degradation pathway and impair the lysosomal homeostasis by downregulating Cathepsin D (Zhang et al., 2018), the reason why damaged lysosomes could not be regenerated was still unknown before this work.

The TFEB, a major regulator of lysosomal biosynthesis, is coordinated by driving autophagy and expression of lysosomal genes (Settembre et al., 2011). However, in this work, we found that a high concentration of midazolam-disturbed lysosomal homeostasis was accompanied by TFEB nuclear translocation failure. Furthermore, we proved that midazolam could also prevent starvation-triggered TFEB transportation. And in this case, damaged lysosome could not have been cleared and regenerated (Nixon and Cataldo, 2006; Settembre et al., 2012), which may account for the irreparability of lysosomal homeostasis. However, the exact relationship between TFEB and disturbed lysosomal homeostasis needs to be further explored.

The impaired autolysosomal function has been reported in sevoflurane-exposed AD mice, and it may be involved in the acceleration of the AD pathological process by sevoflurane (Geng et al., 2018). In this work, midazolam disturbed the lysosomal homeostasis and increased the levels of AD-related proteins, C83, C99 and Aβ40 and Aβ42. Although lysosome is the important organ for Aβ degradation, and enhanced lysosomal function could alleviate AD pathology and improve cognitive function of AD mouse (Nixon et al., 2000; Zhang et al., 2009), whether disturbed lysosomal homeostasis accounts for midazolam-induced accumulation of Aβ still requires verification. In addition, this work is carried out *in vitro* since *in vitro* cell experiments are unilateral and the pathological process cannot be completely simulated *in vivo*. Thus, the effect and mechanism of action of midazolam in AD should be further investigated *in vivo*. Studies have shown that TFEB enhances flux through lysosomal degradative pathways to induce APP degradation and reduce Aβ generation. Activation of TFEB in neurons is an effective strategy to attenuate Aβ generation and attenuate amyloid plaque deposition in AD (Xiao et al., 2015). In this work, we found that midazolam disrupted the lysosomal homeostasis, prevented TFEB from entering the nucleus, and increased accumulation of Aβ in the cells. Therefore, overexpression of TFEB probably reduced the accumulation of Aβ by midazolam, which needs further study. In summary, midazolam disturbed the homeostasis of lysosomes and prevented the TFEB transport to the nucleus. Midazolam also enhanced the accumulation of AD-related proteins C83, C99, Aβ40 and Aβ42. The results indicated the risk of accelerating the pathogenesis of AD by midazolam and suggested that TFEB might be a candidate target for reduction of midazolam-dependent neurotoxicity.

## Author Contributions

DC and QT designed and conducted the study, analyzed and interpreted the data and wrote the manuscript. QZ participated in the work of additional figures 1 and 2 in the later period and participated in the revision of the article. JZ, XH and PF helped conduct the study. WJ provided us with a strain of cells. XL conceived and designed the study, analyzed and interpreted the data, and drafted and critically revised the manuscript.

## Conflict of Interest Statement

The authors declare that the research was conducted in the absence of any commercial or financial relationships that could be construed as a potential conflict of interest.
