# Supplementary material for: TFEB Probably Involved in Midazolam-Disturbed Lysosomal Homeostasis and Its Induced β-Amyloid Accumulation
Source: Front Hum Neurosci. 2019 May 21;13:108. doi: 10.3389/fnhum.2019.00108 (PMC6536689; doi:10.3389/fnhum.2019.00108)
Supplement: Supplementary file 1 [file Presentation_1.pdf]

Different concentrations of midazolam have similar damage to lysosomal homeostasis

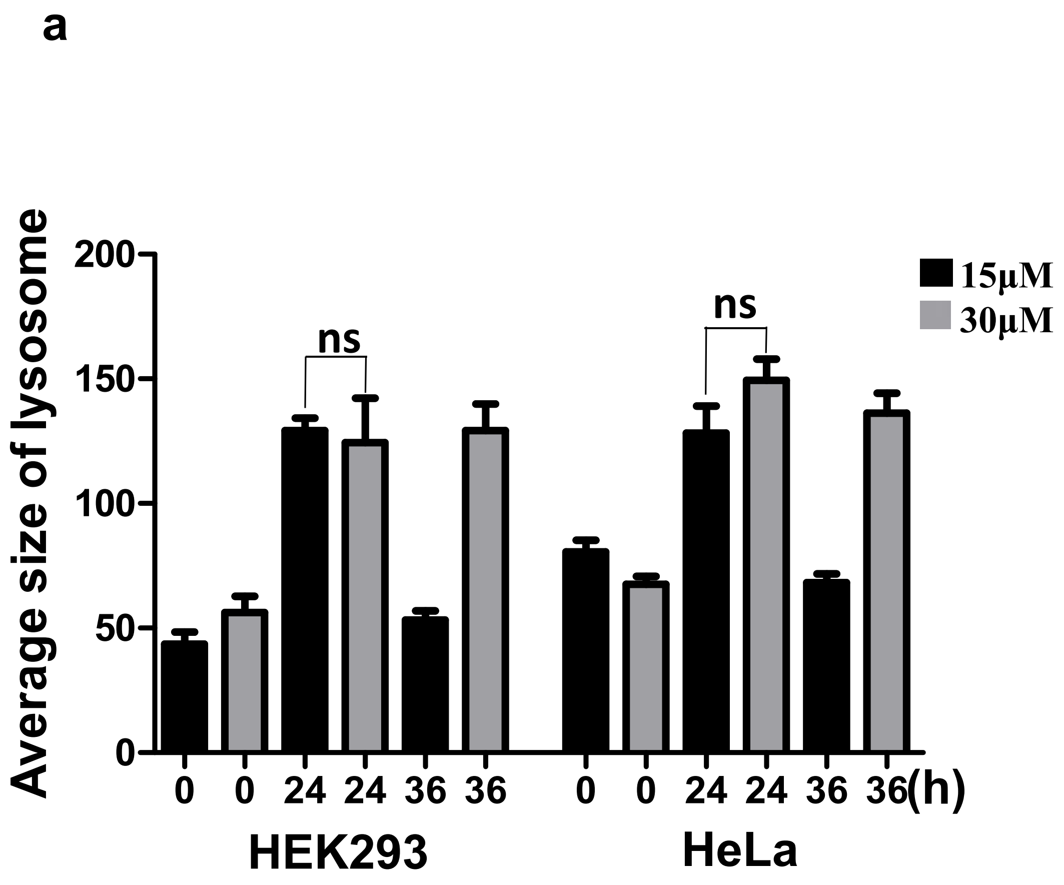

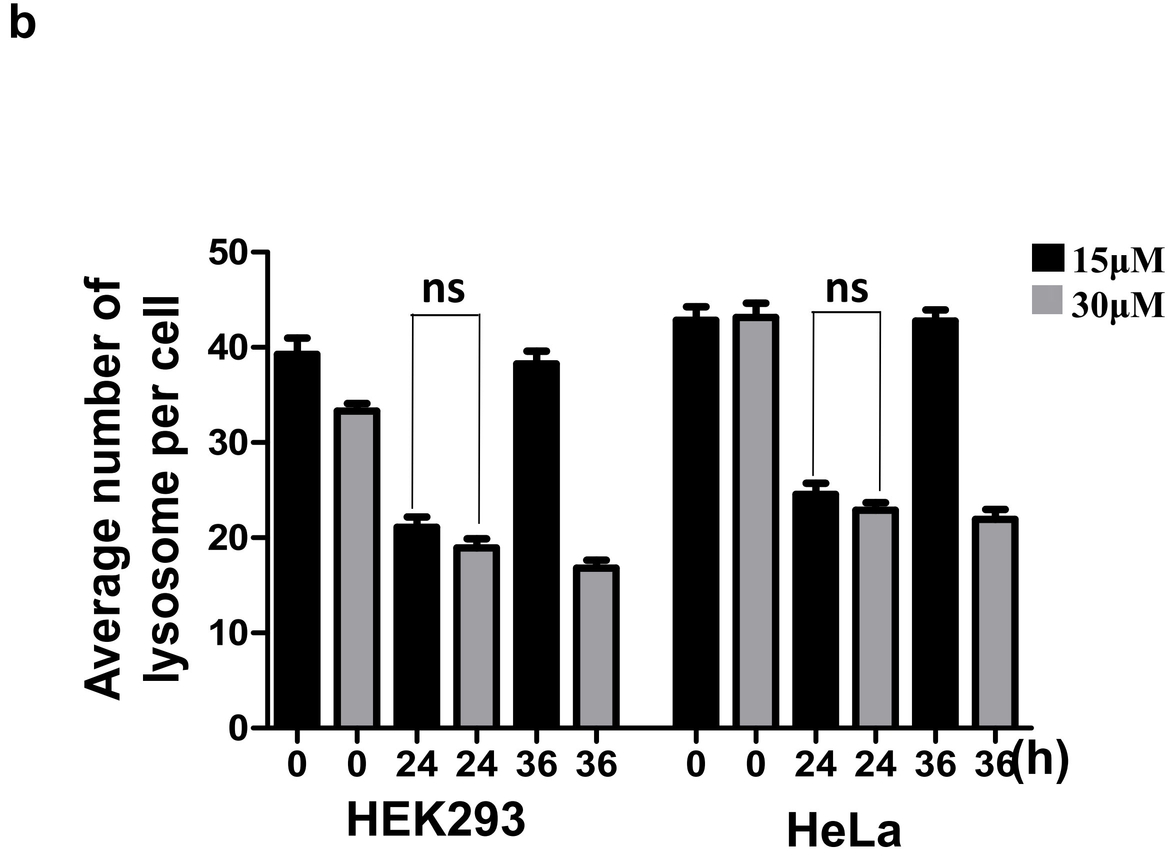

supplementary figure 1

High concentration of Midazolam impair lysosomal homeostasis in HT22 cells

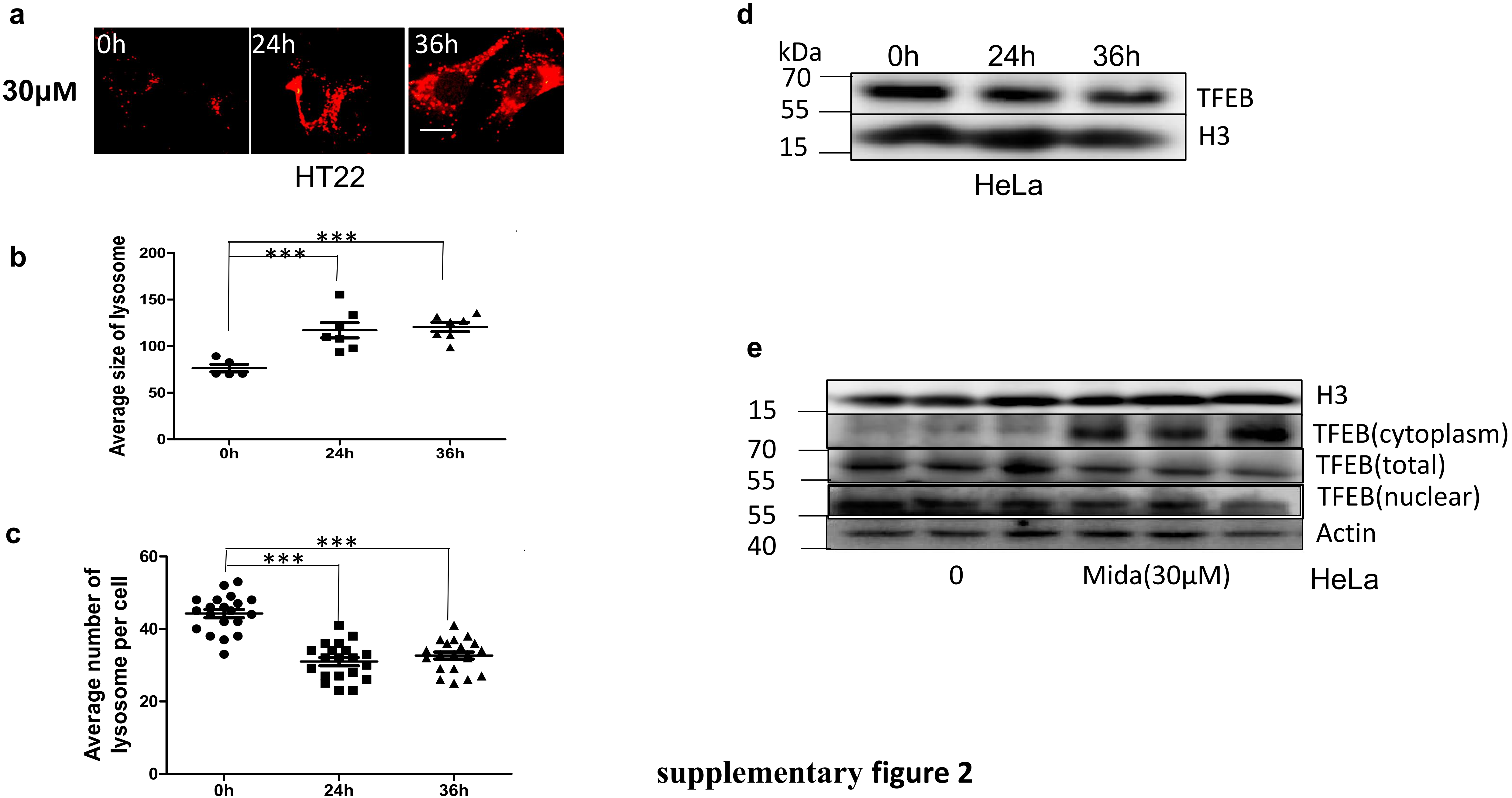

supplementary figure 2

supplementary figure 1. Different concentrations of midazolam have similar damage to lysosomal homeostasis. The size(a) and morphology (b) of lysosomes in HEK293, HeLa cells treated with 15 μM or 30 μM midazolam at 24h. (ns: no significant difference)

Supplementary figure 2. High concentration of Midazolam impair lysosomal homeostasis in HT22 cells. HT22 cells were treated with 30 μM midazolam for 0-36h. (a) Lysosomal staining pattern of HT22 cells treated with 30 μM midazolam at 0 h, 24 h, and 36 h. (b,c) Statistical results of the average size and number of lysosomes in HT22 cells respectively.(d)Western blot results of nuclear TFEB levels in HeLa cells after high concentration of midazolam. (e)Western blot results of TFEB in total, cytoplasmic, and nuclear after treated with 30 μM midazolam.For lysosomal number and lysosomal size statistics, at least 30 cells per goup were counted. Scale bar = 2μm (Mean ± SEM. ***p < 0.001).
